# Morphological, Physiochemical and Thermal Properties of Microcrystalline Cellulose (MCC) Extracted from Bamboo Fiber

**DOI:** 10.3390/molecules25122824

**Published:** 2020-06-18

**Authors:** Masrat Rasheed, Mohammad Jawaid, Zoheb Karim, Luqman Chuah Abdullah

**Affiliations:** 1 Laboratory of Biocomposite Technology, Institute of Tropical Forestry and Forest Products (INTROP), Universiti Putra Malaysia, UPM Serdang 43400, Selangor, Malaysia; masuqayyuum@gmail.com; 2 MoRe Research Ornskoldsvik AB, Box 70, SE-89122 Ornskoldsvik, Sweden; zoheb.karim@gmail.com; 3 Institute of Architecture and Civil Engineering, South Ural State University, 454080 Chelyabinsk, Russia; 4 Department of Chemical and Environmental Engineering, Faculty of Engineering, Universiti Putra Malaysia, UPM Serdang 43400, Selangor, Malaysia; chuah@upm.edu.my

**Keywords:** bamboo, cellulose, microcrystalline cellulose, morphological properties, structural properties, thermal properties

## Abstract

Bamboo fibers are utilized for the production of various structures, building materials, etc. and is of great significance all over the world especially in southeast Asia. In this study, the extraction of microcrystalline cellulose (MCC) was performed using bamboo fibers through acid hydrolysis and subsequently different characterizations were carried out using various advanced techniques. Fourier transform infrared (FTIR) spectroscopy analysis has indicated the removal of lignin from MCC extracted from bamboo pulp. Scanning Electron Microscopy (SEM) revealed rough surface and minor agglomeration of the MCC. Pure MCC, albeit with small quantities of impurities and residues, was obtained, as revealed by Energy Dispersive X-ray (EDX) analysis. X-ray diffraction (XRD) indicates the increase in crystallinity from 62.5% to 82.6%. Furthermore, the isolated MCC has slightly higher crystallinity compared to commercial available MCC (74%). The results of thermal gravimetric analysis (TGA) demonstrate better thermal stability of isolated MCC compared to its starting material (Bamboo fibers). Thus, the isolated MCC might be used as a reinforcing element for the production of green composites and it can also be utilized as a starting material for the production of crystalline nanocellulose in future.

## 1. Introduction

The rapid industrial growth that we are witnessing today has led to an ever-increasing demand for different nano/micro functional and structural materials in various industries for applied research and developments. The modern industrial societies are increasingly extravagant in the use of nano/micro materials for various advance applications. Advanced materials generally have superior properties to conventional materials available, thus they could simply outperform conventional materials in terms of their properties and applications [1,2]. Advanced materials have been extensively used in various high-tech industries, like medical, aerospace, automotive, power sector etc. These materials are normally expensive and small in quantity due to lack of setup used for the up scaling and high production cost. Demand of these products is estimated to grow further and according to a report of materials’ market, the demand is estimated to reach US $102.48 billion by year 2024. There have been intensive efforts to convert existing raw materials into useful functionally and structurally active materials. In parallel, various efforts have also been employed in the utilization of available raw materials for the production of these smart materials. With rapid industrial growth causing an increase in usage of different types of materials, there is an unabated deterioration in our environment. The poisoning of soil-fertility due to non-biodegradability of materials disposed in the soil is continuously adding pollution load to the surrounding environment. Non-biodegradable materials disposed in the soil contribute to reduced soil-fertility by affecting soil properties. Such deteriorating conditions of the soil lead to an alarming sign for the living creatures. Hence, an increasing demand could easily be seen for smart green materials that could replace the fossil-based materials for better human society. In this regard, various research activities have focused on eco-friendly materials that could be used for various applications without compromising the production cost, scalability and final properties. There is a considerable pressure to focus on renewable bio-resources for future materials production. The steps taken towards this direction will lead towards green and eco-friendly science and technology. One important step in this direction is to make use of natural redeemable advanced materials for development as well as fabrication of polymer composites [3,4,5].

In this context, cellulose is one important polymer, which could be tuned according to the requirements and could also be used as functional as well as structural material for the production of valuable composites. It is the most abundant organic compound found on earth that has long been a major reliable renewable source of materials [6,7]. Furthermore, it exists naturally, has low cost, is biodegradable, is a low-density compound, and fits best in the field of renewability. Cellulose, representing about 1.5 × 1012 tons of the total annual biomass production, is considered as a virtually inexhaustible source of naturally available raw materials. It possesses an interesting structure comprising of a linear carbohydrate polymer and long chains of β-d-glucopyranose units further joined by β-1,4-glycosidic linkage [8]. Besides, it has some very important properties that include renewability, biocompatibility, and biodegradability. It also possesses broad chemical-modifying capacity [9].

Cellulose can be obtained from many natural resources, for example, wood, plant, bacteria, and algae. Through hydrolysis process, cellulose can be converted to microcrystalline form known as Microcrystalline Cellulose (MCC) or nanocrystalline form known as Nanocrystalline Cellulose (NCC).

MCC, one of the cellulose derivates, is a naturally occurring particle. It is a fine, odourless white, crystalline powder and possesses important characteristics that include non-toxicity, biocompatibility, biodegradability, high mechanical strength, large surface area, and low density, etc. [10,11]. Due to these properties, it has received great deal of attentions over the last few decades and has been used in different industries. It has especially been used in food, cosmetic and medical industries such as a binder and filler in food, medical tablets etc. Furthermore, it has been used as a reinforcing agent in the development of polymer composites. MCC has also been utilized as a suspension stabilizer, a water retainer, a viscosity regulator, and emulsifier in pastes and creams [6,7,12,13]. Usually, purified and partially depolymerized cellulose is used to obtain MCC. The conventional preparation process involves treating of alpha cellulose with excessive amount of mineral acids. Different cellulose based resources that have been used for MCC preparation include oil palm biomass residue [8,14], rice husk [15], cotton wool [12], mangosteen [16], roselle [17], etc.

In this study, a very smooth, well known, highly tunable and straightforward approach was chosen for the isolation of MCC from bamboo fibers. Bamboo is by far the most important among the natural fiber plants because of its rapid growth rate and universality. Bamboo is a group of perennial evergreens in the true grass family Poaceae, subfamily Bambusoideae, tribe Bambuseae. More than 1,450 bamboo species from 70 genera are found in diverse climates-from cold mountains to hot tropical regions [18,19]. Bamboo fibers have been used in various industries that include textile, automotive, medicine, food industry, etc. [20]. Bamboo is considered an ideal raw material for the production of sustainable building materials in the construction sector accounting for almost 30% to 40% of the global annual bamboo consumption [21]. The reason to choose bamboo fibers as starting materials is because of their strong durability, stability and tenacity and sometimes antibacterial activity (depending on their isolation process). Thus, authors are assuming that isolated MCC from bamboo might have some novel obtained properties like high crystallinity, high thermal degradation temperature, narrow particle size distribution, and controlled topography etc. Furthermore, various advanced and sophisticated techniques are utilized for the characterization of materials. For size and shape determination, micro-/nano- morphology of isolated MCC was observed by Scanning Electron Microscopy (SEM); change in chemical behaviours was analysed by Fourier transform infrared (FT-IR); X-ray diffraction (XRD) was used for the measurement of change in crystallinity; and thermal stability of MCC was analyzed using thermogravimetric analysis (TGA) instrument.

## 2. Characterization

Various sophisticated techniques have been used for the characterization of intermediate materials and final product, MCC.

### 2.1. Chemical Changes Analysis during Procedure

FTIR was carried out to understand chemical changes during the production of MCC. Perkin-Elmer 1600 Infrared spectrometer was used for the detection of various functional groups introduced during the isolation procedure. The spectra are collected by the spectrometer with 32 running scans at a resolution of 4 cm^−1^ for each sample within 650–4000 cm^−1^ range. The “find peak tool” functionality of Nicolet software (OMNIC, version 5.01) was used to determine the positions of significant transmittance peaks at a particular wave number.

### 2.2. Morphological, Particle Size and Elemental Analysis

To determine morphology of MCC samples, scanning electron microscope (SEM) (Hitachi Model S-3400N) was used. SEM model comes laced with energy dispersive X-ray (EDX) equipment, which has a 15kV accelerating voltage. To minimize the charging effect, the MCC samples were gold sputtered and subsequently the samples were observed. EDX diffraction was used for identification of elemental composition of the MCC samples. The analysis of the particle size of MCC samples was performed using Mastersizer 2000 particle size analyser.

### 2.3. Crystallinity Analysis

X-ray diffraction (XRD) is a very powerful technique which is widely used for characterizing crystalline materials. In this study, XRD patterns were recorded by SIEMENS D5000 X-ray diffractometer using Ni-filtered Cu K-alpha radiation using an angular incidence of 5° to 40°. The percentage of crystallinity index (Crl) was calculated using Equation (1):

(1) $$Crl\left(\%\right)=\frac{I002-Iam}{I002}$$

where *I*002 represents the peak intensity corresponding to the crystalline domain (20° to 19.0°), and Iam represents the peak intensity corresponding to the amorphous domain (2° to 22.6°).

### 2.4. Thermal Analysis

Thermal gravimetric analysis (TGA) was performed to analyse the thermal properties of MCC using a Q500 Thermogravimetric analyser (TA Instrument, New Castle, DE, USA). The samples, weighing about 6 mg, were subjected to pyrolysis under nitrogen gas (N2) within 30 °C to 900 °C temperature with 20 °C/minute heating rate.

## 3. Results and Discussion

### 3.1. Visual Analysis, Yield and Physiochemical Categorization

In this study, a well-known procedure, acid hydrolysis, was followed for the isolation of MCC from bamboo. It is worth mentioning that the opted procedure is easily scalable and by changing the concentration of used solvents/chemicals, degree of polymerization, crystallinity of MCC, particle size distribution, and thermal properties etc. it could easily be controlled. The chemical composition of used bamboo fibers is mentioned in Table 1. Furthermore, Table 1 also expresses the terminology used in this article for notation of various intermediate (cooked and bleached pulp) and final material (microcrystalline cellulose). The yield of MCC was ≈ 80% as reported in Table 1. In a study, reported by Ahmed et al. 2016, percentage yield of MCC isolated from rice husk was 60.24% when 1 M HCl was used in the isolation procedure. Furthermore, yield was increased to 69.23% when 2 M HCl was used in the acid hydrolysis step. In the current study, a high percentage yield of MCC was recorded compared to a previously reported study. A high concentration of HCl (2.5 M) in the current study was used to breakdown highly oriented bundles present in the bamboo fibers. In our previous study, MCC were isolated from oil palm, yield of isolated MCC was 60–70% depending on used acid concentration [22].

**Figure 1:**
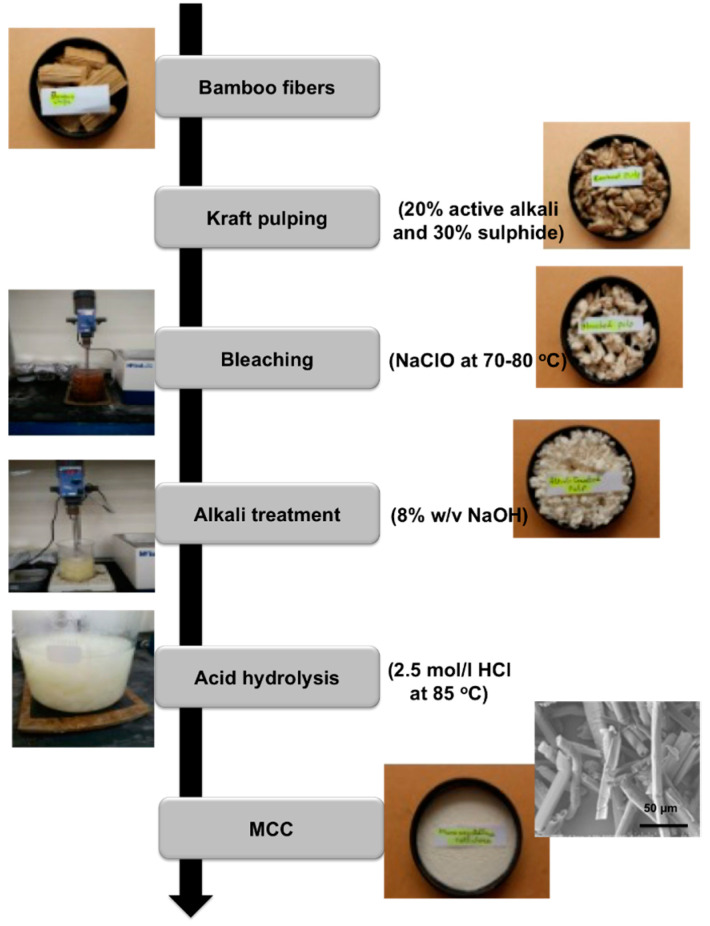
Isolation of MCC from bamboo fibers.

Figure 1 provides various steps used for the isolation of MCC from bamboo fibers. Kraft pulping using active alkali treatment followed by bleaching with NaClO at 70–80 °C was performed. The second last step was alkali treatment using 8% NaOH and later acid hydrolysis was performed using 2.5 mole/l of HCl at 85 °C. Images shown on the left indicate various processing steps used for the isolation of MCC. Intermediate and final products have been shown in the right of the flowchart. A visual expression of isolated MCC is shown in the last image, and SEM image is also mentioned to see the possible morphology. The highest percentage of cellulose (54.61%) was analysed in the bamboo fiber used for the isolation of MCC. First, the craft pulping was performed using alkali and sulphide as shown in Figure 1**,** change is color (brown to pure white) after every applied step indicates the separation of various hemicellulose, lignin, and other impurities from fibers. A pure white isolated MCC is shown in Figure 1.

### 3.2. Analysis of Micro/Nano-Structured Morphology

**Figure 2:**
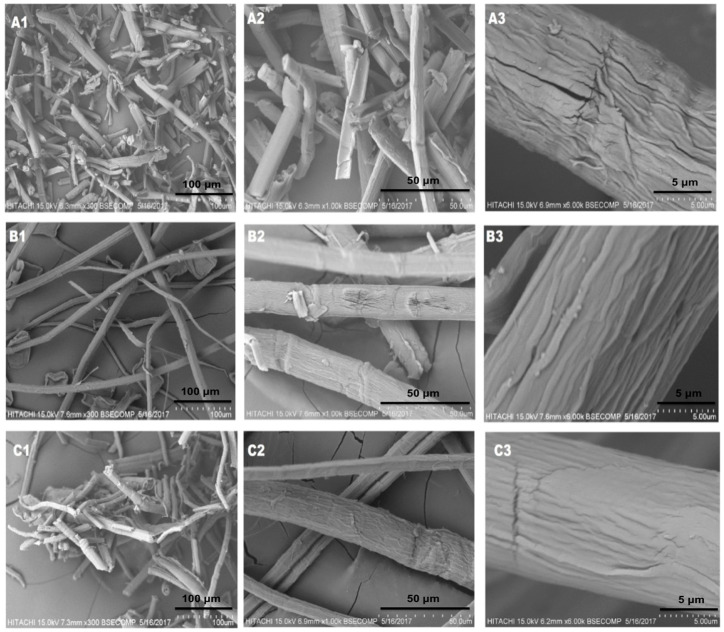
SEM images of isolated MCC (**A**), bleached pulp (**B**) and cooked pulp (**C**). All images are captured at the acceleration voltage of 15 kV as reported in the methods section. Samples were coated with gold before capturing images.

Morphology of isolated MCC and intermediates (cooked and bleached pulp) have been reported in Figure 2. At low magnification, all fibers are in the form of bundles. Various cross-interlocking points/junctions can be seen (Figure 2B1,C1). In the case of MCC (Figure 2A1), a small length and diameter of MCC could be seen, which indicates the removal of lignin, hemicellulose and other impurities during the isolation procedure. It was further visualized that MCC shows individualized fibers having rod like morphology (Figure 2A1–A3). This can be attributed to alkaline and bleaching treatments where the plant’s components get dissolved into well separated fibrous strands [24]. Meanwhile, observation of cooked-pulp Figure 2C1–C3 shows a smooth and clear surface that can be attributed to the partial removal of hemicellulose and lignin [22,25]. When comparing against C-pulp, MCC exhibited non-uniform morphology of micro-sized fibrils having rougher surface (Figure 2A3). This is explained by the structural disintegration of fibrous strands into smaller size microcrystallites during exposure to the HCl treatment, which hydrolytically cleaved the glycosidic bonds of cellulose [26]. According to similar morphological topographies reported in previous studies [27], the rough surface of MCC is affected by the acid hydrolysis, which is favourable for the isolation of nanocrystals. From the observations, MCC also revealed a less aggregated and narrower microstructure compared to C-Pulp and B-Pulp, which exhibited a more disrupted, cracked and aggregated microstructure. This was probably caused by differences in cellulose materials and chemical treatment conditions [28]. The long microfibrillar structure of MCC with a presumably high aspect ratio makes it suitable to be used in the production of high tensile strength biocomposite products.

### 3.3. FTIR Analysis of Intermediate and Final Products

**Figure 3:**
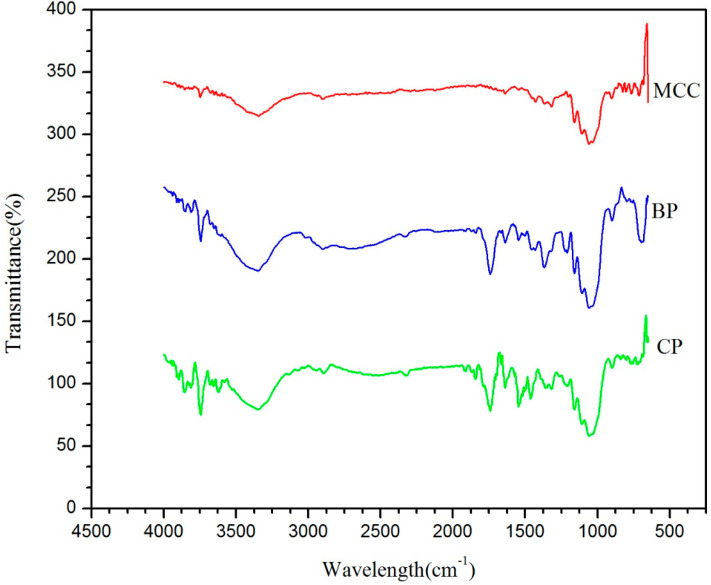
FTIR spectra of MCC, B-PULP, and C-PULP samples are shown in this figure. All samples were analysed in the range 4500 cm^−1^ to 500 cm^−1^ of wavelength and the change in functional group intensity was recorded and reported in the results section.

To study the conformational and physicochemical properties of polysaccharides, FTIR analysis is considered as one of the powerful tools. FTIR spectra was recorded for all the samples in order to observe the change in the peak frequencies of various functional groups. The chemical composition of all the samples does not show marked change as the samples go through the extraction process. The spectra were carried out from 4000 cm^−1^ to 500 cm^−1^ in order to understand the change in the chemistry of various samples during the isolation of MCC from bamboo fibers as shown in Figure 3. The broad bands in the region of 3343 cm^−1^ in the case of cooked pulp and bleached pulp is attributed to the O−H stretching vibrations and the same O−H peak showed a red shift in the case of MCC at 3338 cm^−1^ while the peak frequencies at 2900 cm^−1^ and 2905 cm^−1^ correspond to C−H stretching vibrations. Furthermore, the MCC spectra shows almost the disappearance of characteristic C−O stretching vibration at 1722.16 cm^−1^ and 1533 cm^−1^, which corresponds to the acetyl and uronic ester groups of hemicelluloses as well as the ester linkage of the carboxyl groups of lignin and to the C=C vibration in lignin respectively. Additionally, significant peaks at 896 cm^−1^ (β-glycosidic bonds bending), 1050 cm^−1^ (C−OH stretching), and 1160 cm^−1^ (C−O−C glycoside bonds symmetrical) represent unique fingerprint regions of all the processed samples.

### 3.4. Calculation of Crystallinity Using XRD

**Figure 4:**
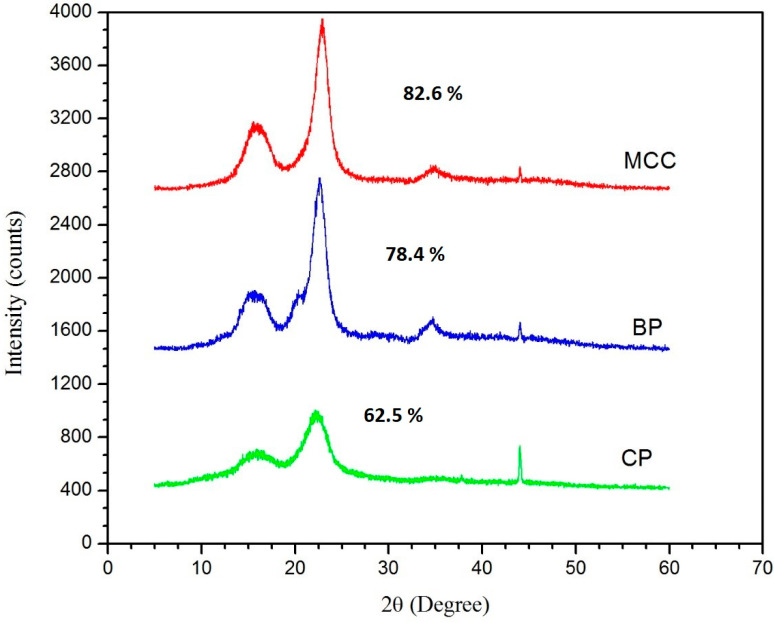
X-ray diffractograms of MCC, B-pulp, and C-PULP samples were taken as reported in the methods section. Furthermore, equation 1 was taken into account for the calculation of crystallinity of intermediate (C-Pulp and B-Pulp) and final isolated sample, MCC.

XRD pattern of CP, BP and MCC are presented in Figure 4, and the calculated crystallinity indexes are shown in image. The highest crystallinity index was recorded for MCC (82.6%), bleached pulp was in the middle range (78.4%), and cooked pulp was lowest (62.5%). The major diffraction peaks for isolated products were observed at around 16.1°, 22.8° and 44.2°, which corresponds to the crystallographic planes of (110), (200) and (004), respectively [27,29]. The peaks at 16.1° and 22.8° have been more sharpened for MCC but in the case of BP, sharpening of the peak could be seen in the middle of MCC and CP. A very broad peak was observed in the case of CP. It has also been seen that the intensity of the peak at 42.2° decreased in order of the isolation process of MCC. A very intense peak was observed for CP, but in parallel the same peak seems to be diminished for MCC. The highest crystallinity index obtained for MCC is attributed to the decrease in its amorphous regions. The amorphous regions from CP to MCC get removed as a result of acid hydrolysis, which prompts the hydrolytic cleavage of glycosidic bonds, ultimately releasing individual crystallites [30]. The reported results are also in agreement with results of [17], where Roselle fibers were used for the isolation of MCC and further compared to commercial MCC. Seventy-eight percent crystallinity was reported for isolated MCC in [17], thus, MCC isolated in the current study has higher crystallinity index (82%) compared to the previous study.

### 3.5. EDX Analysis

**Figure 5:**
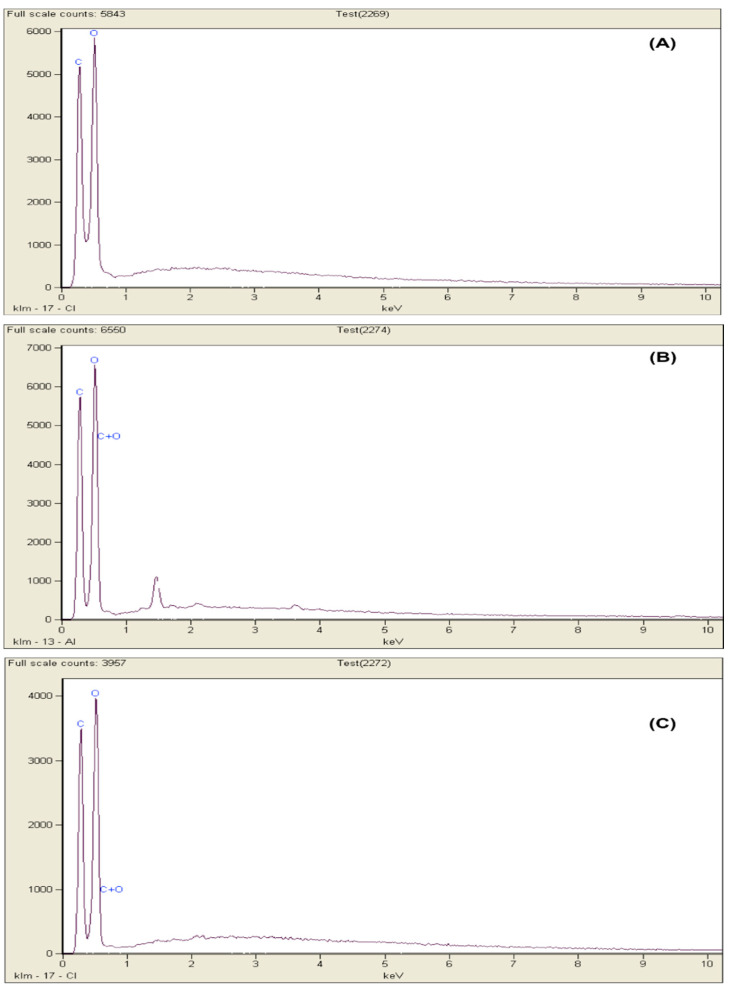
Energy-dispersive X-ray diffraction (EDX) spectra of (**A**) MCC, (**B**) B-Pulp and (**C**) C-Pulp isolated from bamboo fibers.

The EDX spectra for C-pulp, B-pulp and MCC are presented in Figure 5. All EDX spectra exhibit prominent peaks for carbon and oxygen as the main elements in their compositions as expected, correlating well with the distinctive characteristics of cellulose [17,28]. The carbon percentage for C-pulp, B-pulp and MCC is 41.98%, 39.15% and 31.34%, respectively, whereas the oxygen percentage for C-pulp, B-pulp and MCC is 57.96%, 62.15% and 67.37%, respectively. It is observed that the oxygen content for MCC becomes slightly more intense after the chemical treatment of B-pulp. This was due to the delignification process, which results in extraction of the highly purified cellulose with decreased silica impurities and lower phosphorus contents [7,31]. Sodium and chlorine elements were also slightly detected during the chemical treatment processes. Similar spectra have been reported during the preparation of cellulose MCC from roselle fiber by [17]. In addition, close values of carbon and oxygen percentages for B-pulp and MCC were observed. Thus, verifying the elemental compositions of B-pulp and MCC.

### 3.6. Analysis of Thermal Properties

**Figure 6:**
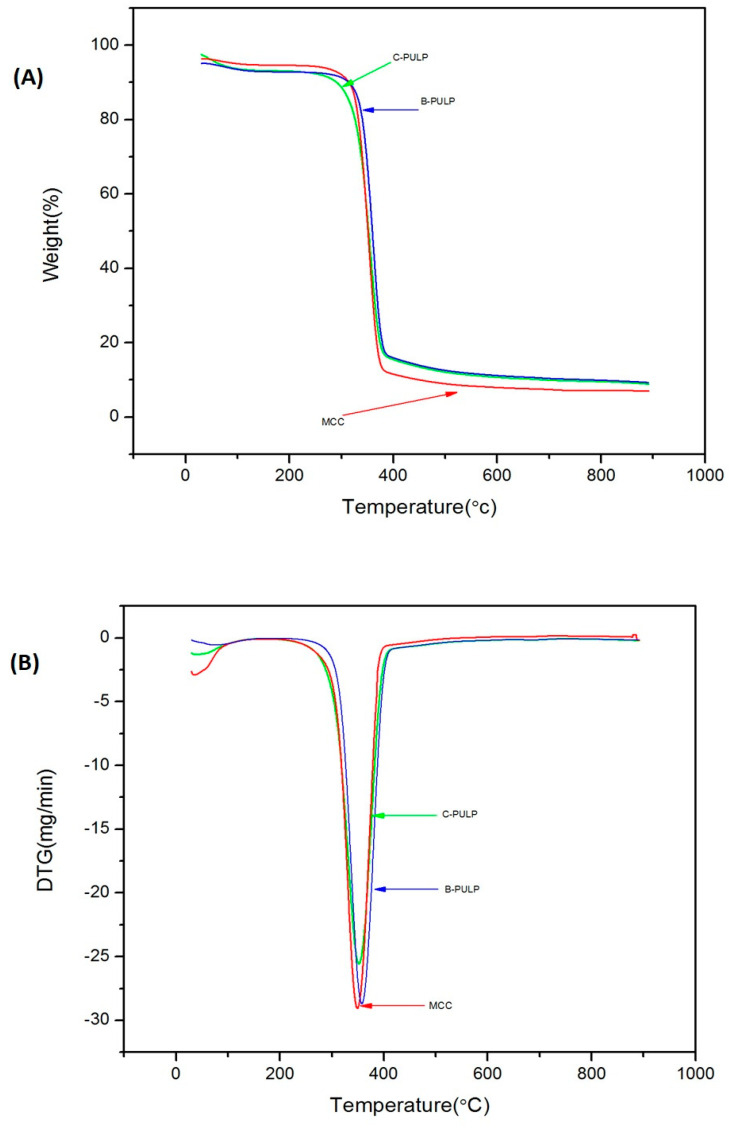
TGA curves of B-Pulp, C-Pulp and MCC samples (**A**) and DTG curves of B-Pulp, C-Pulp and MCC samples (**B**). Analysis of thermal properties was performed as given in the methods section.

The determination of thermal stability is crucial for applications where the processing temperature is higher especially in the production of biocomposites [32,33]. The thermogravimetric analysis (TGA) and derivative thermogram (DTG) curves for C-pulp, B-pulp and MCC are shown in Figure 6A. Table 2 demonstrates the TGA initial and final decomposition temperatures of C-pulp, B-pulp and MCC samples. The thermal degradation mainly occurs in two stages. In the first stage, the initial weight loss as a result of the evaporation of water and other volatile compounds within the samples occurs in the range of 35–305 °C [27,34,35]. The weight loss during this stage was noted to be 13% for C-pulp, 12% B-pulp and 10% for MCC, showing the hydrophilic nature of C-pulp and B-pulp more than for MCC. In C-Pulp and B-pulp due to presence of more amorphous regions, providing more accessibility to water molecules due to the presence of the more non-substituted hydroxyl group, thus the more crystallinity, the lesser the water absorption is. Cellulose decomposition in C-pulp begins at 290 °C, for B-pulp it begins at 314 °C, while for MCC it occurs at 315 °C. This indicates a slightly higher degree of molecular arrangement of MCC, that starts degradation at a higher temperature, and therefore, requires high heat energy for degradation [25]. However, because there is no significant difference between the decomposition temperatures, thus no final conclusion about MCC having a higher degree of molecular arrangement can be made based on the given results. The final temperature of C-pulp occurs at 695 °C; in B-pulp, it is noted at 698 °C and for MCC at 450 °C. The degradation of cellulosic components starts at 150 °C and lasts up to 385 °C, when the decarboxylation, depolymerization and decomposition occur in cellulose and hemicellulose fragments. Biomass is subjected to aromatization, decomposition, combustion, lignin pyrolysis, and char residue formation beyond the temperature of 385 °C [35,36]. The maximum weight loss for C-pulp, B-pulp and MCC occurred at 355 °C, 357 °C and 353 °C respectively. Meanwhile, the char residue weight for C-pulp is 8.4%, for B-pulp is 9.4%, and for MCC is 6.9%. Among the three samples, the char residue is the lowest in MCC, which indicates the high purity of the cellulose in MCC [37]. The high residual weight of C-pulp was mainly due to presence of flame retardation compounds that result in char formation [38,39]. The peak temperature for MCC in our study was found to be 353 °C, which was greater than those of MCC extracted from roselle fiber (340 °C), as reported by [17] and 339 °C for the MCC extracted by [40].

## 4. Materials and Methods

### 4.1. Materials 

Bamboo chips used in this study were provided by INTROP, UPM, Serdang, Malaysia. The initial moisture content of the bamboo chips was 8.3%. The other materials like sulphide, sodium hypochloride (NaClO), hydrochloric acid (HCl) and sodium hydroxide (NaOH) were purchased from Sigma Aldrich, Saint Louis, MO, USA. All chemicals used in this study were laboratory graded and were used as procured.

### 4.2. Experiments

The steps involved in the isolation of MCC from bamboo fibers are given Figure 1. Various steps used for the isolation MCC are Kraft pulping, bleaching, alkali treatment, and acid hydrolysis as discussed below in detail.

#### 4.2.1. Kraft Pulping

Rotary digester having a capacity of 4 L was used for kraft pulping. The 20% of active alkali and 30% of sulphide, in the ratio of 1:7, was used in the process. Cooking was performed at 170 °C for 3 h. The utilized pressure was about 12–14 bar. The pulp was washed and screened using Somerville Shive content analyser and the black liquor was discarded. After that, the pulp was kept in an oven for 24 h at a temperature of 60 °C, resulting in dried pulp.

#### 4.2.2. Bleaching

Dried cooked pulp obtained from kraft pulping was subjected to bleaching process to separate lignin and hemicellulose using sodium hypochloride (NaClO). The process was carried out for 1 h at 70–80 °C. The pH of the solution was 12.0. Acetic acid was regularly added during the bleaching step and constantly stirred until its pH reached 4. Obtained bleached fiber was filtered and washed twice with distilled H_2_O to obtain white-yellowish fibre. Afterwards, it was dried in an oven at 60 °C for 24 h.

#### 4.2.3. Alkali Treatment

The bleached fibers were treated with 8% (*w*/*v*) sodium hydroxide (NaOH) solution (1:50, g/mL^−1^). Reaction procedure was carried out at room temperature for 30 min. Obtained pulp (alkali treated) was filtrated and washed followed by drying in an oven at 60 °C for 24 hrs.

#### 4.2.4. Acid Hydrolysis

Acid hydrolysis is a well-known chemical process for removing the amorphous regions of cellulosic materials. In this process, strong acids like sulphuric acid, hydrochloric acid (HCl), etc. are used for dissolving the unlikely parts of cellulosic fibers. In this study, the fiber (alkali treated) was treated with 2.5 mol/L HCl. Hydrolysis process was carried out at 85 °C for 30 min. and the solid-to-liquor ratio being used was 1:30 (g/mL^−1^). Hydrolysis was performed under agitation using a constant stirring speed. The obtained product was made to cool by keeping it at ambient temperature followed by filtration and washing to make pH neutral. The product obtained was dried in a vacuum at 80 °C until constant weight was achieved. Obtained white power was stored and denoted as MCC in the article.

## 5. Conclusions

This study revealed that bamboo fiber has the potential to be used as an alternative for the extraction of MCC by following processes that include bleaching, alkaline treatment and acid hydrolysis. FTIR spectra indicated that there was no change in the chemical structure of cellulosic components. Moreover, the chemical treatments have caused a significant amount of lignin removal from bamboo fiber. The SEM that provides the morphological analysis indicates that a rough rod-like and lighter structure of MCC were obtained after bleaching and acid hydrolysis of bamboo pulp. The prepared MCC also exhibited a high crystallinity index of 82.6%, which endorses its suitability for application in packaging materials. Based on the TGA analysis, MCC displayed greater thermal stability, confirming its suitability for application in polymeric composites of higher processing temperature. The MCC extracted in this research can find application in biocomposite packaging materials.

## Figures and Tables

**Table 1:** Chemical composition of used bamboo fibers used and terminologies used for the intermediate and final materials [23].

Chemical Composition (%)		Coding of Materials Used in This Article		MCC Yield (%)
Cellulose	54.61	Cooked pulp	C-Pulp	80
Hemicellulose	6.85	Bleached pulp	B-Pulp	
Lignin	20.85	Microcrystalline cellulose	MCC	
Others	17.69			

**Table 2:** Thermal properties of cooked pulp, bleached pulp and isolated MCC.

Samples	T_initial_ (°C) ^a^	T_final_ (°C) ^b^	W_residue_ (%) ^c^
C-Pulp	290	695	8.4
B-pulp	314	698	9.4
MCC	315	450	6.9

^a^ TGA initial decomposition temperature, ^b^ TGA final decomposition temperature, ^c^ TGA char residue weight.
